# AnEEG: leveraging deep learning for effective artifact removal in EEG data

**DOI:** 10.1038/s41598-024-75091-z

**Published:** 2024-10-16

**Authors:** Bhabesh Kalita, Nabamita Deb, Daisy Das

**Affiliations:** Department of Information Technology, Gauhati University, Guwahati, Assam 781014 India

**Keywords:** Artifacts, Generative adversarial network, Deep learning, EEG, LSTM, and GAN, Neuroscience, Cognitive neuroscience, Computational biology and bioinformatics, Computer science, Information technology

## Abstract

In neuroscience and clinical diagnostics, electroencephalography (EEG) is a crucial instrument for capturing neural activity. However, this signal is polluted by different artifacts like muscle activity, eye blinks, environmental interference, etc., which makes it more difficult to retrieve important information from the signal. Deep learning methods have demonstrated the potential to lower these artifacts and enhance the EEG’s quality in recent years. In this work, a novel deep learning method,“AnEEG” is presented for eliminating artifacts from EEG signal. The quantitative matrices NMSE, RMSE, CC, SNR and SAR are calculated to confirm the effectiveness of the proposed model. Through this process, it was found that the suggested model outperformed wavelet decomposition techniques. The model achieves lower NMSE and RMSE values, which indicates better agreement with the original signal. Achieving higher CC values means stronger linear agreement with the ground truth signals. Additionally, the model shows improvements in both SNR and SAR values. Overall, this suggested approach showcases promising results in improving the quality of EEG data by utilizing deep learning.

## Introduction

One of the most important foundations of neuroscience studies is electroencephalography (EEG), which has produced invaluable insights regarding the vital functions of the human brain. EEG is recorded by capturing the electrical fluctuations of neurons, it does so by using a series of electrodes applied to the scalp to measure voltage fluctuations^1^. The versatility of EEG extends not only to BCI applications but also to clinical diagnostics in neurology, whereby it helps solve mysteries about the brain and diagnose neurological disorders, among many others. Through EEG, people have been able to investigate brain function, detect neurologic disorders, and even delve into human cognition depths^2^. However, the occurrence of unwanted artifacts makes the usefulness of EEG data highly problematic. cost-effective, simple, and exceptional temporal resolution is the main advantage of EEG^3^ from the abstract. This explains why it’s employed in several medical and scientific domains. ocular, muscle, powerline noise, and other environmental factors polluted the EEG signal during recordings from the abstract.

Artifacts are mainly separated in 2 categories: biological sources and environmental sources. Biological artifacts are generated by the human body and serve functions like ocular movements, cardiac rhythms, muscular contractions, etc. These biological noise sources can distort the recorded EEG signal, making it difficult to accurately interpret or analyze them. On the other hand, environmental noise originating from outside includes power line interference as well as inadvertent electrode movement. The second source is environmental artifacts generated from external sources like powerline interference, the movement of electrodes, etc. These artifacts also corrupt the structure of EEG data, adding to the complexity of the artifact removal task^4^.

These artifacts render EEG incomprehensible and unreliable but also add complexity to the process of meaningful analysis and diagnosis. Unable to address these artifacts not only complicates the interpretation of brain activity but also has important implications in clinical practice since their wrong presence can lead to misdiagnosis and treatment. Consequently, substantial efforts have been made in the field of EEG signal processing to create reliable artifact removal methods. Typically, analyzing EEG data involves considering signals within the range of 1–100 Hz. This range includes both neuronal signals that are the main and artifact signals, thus making their separation a daunting task. These include eye blinks, which typically have low-frequency components below 4 Hertz, and muscular activity with high-frequency characteristics above 13 Hz, thereby overlapping with genuine frequencies associated with EEGs^5^. As such, it is extremely difficult to separate these artifacts from their sources to maintain the rich neural information contained in an EEG dataset.

Several methods have been proposed to solve this problem and have been improved over the years, but sometimes they may not be effective in solving it^6^. These methods are mainly classified into two main approaches: those that estimate and remove artifacts from reference channels, and those that decompose EEG signals into alternative regions for artifact extraction. These methods include regression-based methods^7^, Blind Source Separation(BSS)^8^, Wavelet Transform, EMD (empirical mode decomposition)^9^, and their hybrid variants. Successfully identifying and eliminating artifacts from EEG-signals is a very important task in both clinical and practical applications. Manually visualizing segments, identifying, and removing the artifact causes significant loss in valuable neural data. Therefore, research needs to develop more sophisticated artifact removal methods, particularly when it comes to physiological artifacts.

Deep learning, a constantly growing technique, has shown its ability to overcome these challenges in recent years^10^. As a type of neural network, Generative Adversarial Networks (GANs) have demonstrated success in creating data that is nearly devoid of artifacts. GAN consists of two main components: a discriminator and a generator. The main job of the discriminator is to differentiate genuine from generated data, and on the other hand, the generator attempts to provide data that is identical to the genuine data^11^. This adversarial learning approach has demonstrated remarkable effectiveness in generating artifact-free EEG signals. This study takes an important step by proposing a new approach to artifact removal techniques that enhances the capabilities of GANs by integrating long-term and short-term memory-LSTM layers. LSTM, a type of recurrent neural network, is effectively capturing temporal dependencies and contextual information, making it well suited for EEG data processing tasks^12^, where temporal dynamics are important.

The primary objective of this work is to remove artifacts from EEG using AnEEG, a LSTM-based GAN deep model. The goal was to enable the GAN to generate pure EEG signals that maintain the original neural activity data by training the model on a variety of datasets containing EEG recordings with various artefacts. In a GAN architecture, the discriminator examines the quality, compares the generated data with ground-truth data, and guides the generator to produce more accurate signals without artifacts.

In the following sections of this paper, the methodology, experiments, and results of the proposed AnEEG model are explained. The effectiveness of the model in removing artifacts under different types of artifacts and recording conditions will also be demonstrated in the following parts. Ultimately, the proposed effort will significantly improve the quality of EEG data, which will increase the reach and significance of neurobiological research and therapeutic applications based on EEG.

## Literature survey

In neuroscience research, removing artifacts from EEG-signals is a very essential task, because artifacts seriously affect the accuracy and reliability of the recorded data. Various approaches have been put forth to deal with this issue, with an emphasis on the creation of sophisticated techniques and clever algorithms for artifact removal. Literature shows that GANs are useful not only as generative models but also as a denoising technique for artifacts. Yang An et al.^13^ described an automatic denoising method for multichannel-EEG signal using a GAN-Generative Adversarial Network. To determine whether or not the filtered EEG can preserve as much of the original effective information, they suggested a new loss function. EEG signal’s range limitation was achieved by utilizing energy threshold-based normalization and sample entropy to identify anomalous signals. For removing noise, they defined a method of using GAN-based blind denoising and the discriminator was used to judge whether the noise was filtered out or not. In this work, they used the HaLT dataset that recorded 12 subjects and 960 trials per participant with six imaginary motion states. Data is collected using 22 electrodes and the data sampling frequency is 200 Hz.

For practical BCI applications, Eoin Brophy et al.^14^proposed a GAN-supervised technique for EEG signal denoising. They created a pipeline that removed artifacts from EEG time series data. In this experiment, the generator was fed noisy EEG data, and the resulting clean EEG signal was compared to it in the discriminator. With one layer fully connected to the output, the generator composed of a two-layered array of long-term short-term memory (LSTM) with 50 units hidden in each layer. The discriminator is a four-layer, one-dimensional convolutional neural network with a sigmoid function. This experiment made use of the open-source PhysioNet motor/imaging dataset. A 64-channel BCI2000 system with a 160 Hz data sample rate is used to record data.

Tian-Jian Luo et al.^15^ developed a technique for reconstruction of EEG signals using GAN with temporal-spatial-frequency loss function and Wasserstein distance. By calculating the mean-squared error from time series and power spectral density features, the loss function recreated the signal. Three distinct datasets with varying sampling rates were used in this experiment to train and assess the networks. The 2018-published Action Observation Dataset was the first dataset, and it was sampled at 250 Hz and 64 channels. The Grasp and Lift (GAL) dataset was the second one. A “Brain Amp” device with thirty two channels, sensitivity, and sampled at 500Hz and 0.1 mv is used to collect the data. The third dataset was the Motor Imagery dataset. Data was collected from 12 subjects using 32 channels, and the sampling rate was 250Hz.

Jin Yin et al.^16^ presented a technique for denoising EEG signals dubbed GCTNet, which included a transformer network and a GAN-guided parallel CNN. The discriminator identified and rectified holistic discrepancies between clean and denoised EEG signals, while the generator composed of parallel CNN blocks and transformer blocks, captured both global and temporal dependencies. To assess their approach, the researchers used one genuine dataset and three semi-simulated datasets. The MIT-BIH Arrhythmia Dataset combined clean ECG signals with EEG segments; the EEG DenoiseNet dataset combined clean EEG segments with EMG and EOG segments; and the semi-simulated EEG/EOG dataset involved linearly mixing EOG artefacts with clean EEG signals. The actual dataset is made up of recordings from a patient with epilepsy that were made for 10 s at a sampling rate of 250 Hz and included 21 channels. Compared to other existing approaches, GCTNet showed a considerable performance improvement, achieving a reduction of 11.15% in relative root mean square error-RRMSE and an improvement of 9.81 in signal-to-noise ratio-SNR.

Phattarapong Sawangjai et al.^17^ developed an Ocular artifact removal framework ‘EEGENet’, based on generative adversarial networks. The algorithm runs under various conditions, i.e., no Eye-movement, vertical Eye-movement, horizontal Eye-movement, and eye blinking. For the training set, state-of-the-art EOG suppression techniques were used to create a clean target EEG signal that could be used as the original truth. The cleaned dataset is used to train the discriminator, and raw EEG data is passed into the Generator. For the data, they used 3 open-source datasets. The first one was the EEG Eye Artefact Dataset, which was pre-processed using a notch filter after data was gathered from 50 subjects. The second dataset, BCI Competition IV2b, has three channels and a 250Hz sampling rate. The Multimodal Signal Dataset was the third dataset. It featured sixty electrodes and a 2500 Hz sampling rate.

Sandhyalati Behera et al.^18^ proposed a machine-learning based approach for eliminating artifacts from EEG signals. To address the challenges posed by nonlinear and non-stationary artifactual signals, they utilize a RVFLN(Random Vector Functional Link Network) model. An exponentially weighted RLS(Recursive Least Squares) algorithm is employed to build the adaptive filter within this novel RVFLN model. To build and validate their model, clean EEG data is sourced from the openly available Mendeley database, while ECG signals, used to create artifacts in the EEG signals, are collected from the Physionet database. This approach aims to effectively remove artifacts and verify the efficacy of the proposed algorithm.

A novel approach to artifact removal was put forth by Zainab Jamil et al.^19^, where they classified EEG fragments based on Eye-movement and then used discrete wavelet transform and independent component analysis to eliminate artifacts. The dataset was obtained under unrestricted conditions, from 29 subjects who engaged in activities such as walking, watching videos, and expressing gestures and facial expressions. EEG signals were processed by extracting thirteen morphological features to identify segments containing Eye-movements. These segments are then denoised without distorting the signal morphology.

Rajdeep Ghosh et al.^20^ provided a dependable technique for automatically identifying and eliminating muscular artifacts and eye blinks from EEG recordings. This method combines a long short-term memory-LSTM with a k closest neighbour (KNN) classifier. A sliding-window method lasting 0.5 s is utilized to find and eliminate artifacts. For every EEG segment, the properties of peak-to-peak amplitude, average rectified value, and variance are determined. Bed segments were identified by KNN classifier and then processed by the LSTM network to eliminate artifacts. The suggested techniques showed outstanding performance in terms of the signal-to-artifact ratio,correlation coefficient, structural similarity, and normalized RMS error, with an accuracy of 97.4% in identifying damaged segments.

Shalini Stalin et al.^21^ suggested an approach for identifying and eliminating major movement artifacts from one channel EEG. “Support Vector Machine(SVM)” was utilized in the detection procedure, and “Ensemble Empirical Mode Decomposition (EEMD)” was utilized to extract signal features. Motion artifacts can be eliminated with the use of canonical-correlation-analysis-CCA. And the remaining motion artifacts were eliminated using the WT-(Wavelet Transform) technique, they employed the “Hawks optimization algorithm (HHO)” to optimize the outcomes.

Sakib Mahmud et al.^22^ proposed the Attention Guided Operational CycleGAN (AGO-CycleGAN), to remove motion artifacts and enhance the quality of corrupted EEG signals. This approach combines PatchGAN-based discriminators with attention-guided Feature Pyramid Network generators that are adjusted bottlenecks, as well as self-generative operational neurons. The model was tested and trained on a single-channel EEG dataset of 23 subjects, using a subject-independent Jackknife cross-validation approach. The method outperformed other techniques and was evaluated through both qualitative and quantitative analyses, employing robust metrics in the temporal and frequency domains.

**Table 1:** Summary of literature survey.

Author/year	Datasets	Methods	Performance	Limitations
^13^/2022	HaLT public dataset	ICA denoising, EMD, wavelet transform filtering, GAN.	CC:0.78 and RMSE: 0.076; compares with artificial methods.	Volatile signal changes do not filter well; GAN struggles with noise-heavy signals, and EMG noise is ignored.
^14^/2022	PhysioNet, EEGdenoiseNet	GAN (LSTM, Conva2D)	RRMSE_temp_: 0.05, CC: 0.89 and RRMSE_spec_: 0.1 for EEGdenoiseNet dataset	Lacks comprehensive investigation into the model’s ability to effectively remove EMG noise.
^15^/2020	AO (2018), GAL (2014) and Motor Imagery dataset (2012)	Wasserstein Generative adversarial networks( temporal-spatial-frequency loss function).	Improved classification accuracy to 67.67% (AO), 73.89% (GAL), and 64.01% (MI).	The CNN models take significant time to build and train, challenging real-time application feasibility without optimization.
^16^/2023	EEGdenoiseNet, MIT-BIH Arrhythmia Dataset, Self.	GAN-guided parallel CNN and transformer network	Achieving an 11.15% reduction in RRMSE and a 9.81% improvement SNR.	Training on semi-simulated data may limit real EEG applicability, focusing on single-channel neglects spatial information in multi-channel data.
^17^/2021	Eye Artifact , BCI Competition IV 2b, Multimodal Signal Dataset	Generative Adversarial Network ( Conva2D)	Achieving RMSE: TD—3.64$\mu$V, FD—0.24pV^2^/Hz, superior to CNN autoencoder.	Focus on ocular artifacts may overlook other types such as muscular artifacts or heart activity, present in certain datasets.
^18^/2023	Mendeley public dataset, Physionet dataset	A Random Vector Functional Link Network, Recursive Least Squares algorithm.	MSE: 0.19 $\mu$V^2^, NMSE: 0.447 $\mu$V^2^, SNR: 68.4 dB; outperforms EMD, EEMD, ICA etc.	Requires knowing the artifact frequency, which can be hard in real-world scenarios.
^19^/2021	Self-collected, obtained in a non-restricted environment.	Removing ocular artifact by classifying eye-movement EEG chunks and using ICA with DWT.	SVM achieves 98.28% accuracy, average sensitivity of 93.55% and specificity of 98.43% compared to unsupervised methods.	ICA requires manual selection of noise components, making it time-consuming.
^20^/2023	Self-collected (32-channel at 1024 Hz)	KNN classifier and a LSTM network.	97.4% accuracy in detecting noise; average CC, SS, SAR, and MSE were 0.69, 0.76, 1.52 dB, and 0.0013, respectively	Focuses only on eye-blinks and muscular artifacts; multiple artifacts within a single window are not considered, its affecting accuracy.
^21^/2021	PhysioNet-motion artifact	SVM, with EEMD for feature extraction, CCA for removal, WT, and optimization using HHO.	EEMD-CCA-SWT significantly reduced RMSE, and improved DSNR, coherence, and PSD values compare to EEMD-CCA.	Focus primarily on motion artifact detection and removal.
^22^/2024	fNIRS and EEG Data from PhysioNet repository	AttentionGuided Operational CycleGAN (AGO-CycleGAN), a CycleGAN-based framework	Achieves 26.497 dB increase SNR, 87.2% enhancement in temporal correlation, and 93.5% improvement in spectral correlation.	Limited to single-channel motion artifact restoration, neglecting physiological artifacts due to absence of ground truth labels.
^23^/2024	Physionet public dataset	Decomposed into IMFs, suppressed with PCA, noisy IMF sections removed based on variance measure	EWT-based denoising outperformed EWT-PCA, with average SNR of 28.26 dB and 55.00% $\eta$ compared to 26.78 dB and 52.92% $\eta$, respectively.	IMFs derived from EMD may inaccurately capture signal structure, particularly in complex noise, leading to incomplete artifact removal.
^24^/2023	Motor imagery brain-computer interface dataset	Forward-Backward Low-Pass Filter, Discrete Wavelet Transform and Adaptive Bi-Orthogonal Wavelet.	FBLPF-ABOW achives highest F1 value compared to SSA-ANC, VME-DWT, FBSE-EWT-LPATV, ITMS, SSA-Kmeans etc..	Fixed-length window for blink artifact detection may miss short-duration artifacts, reducing effectiveness.
^25^/2023	EEGdenoiseNet Benchmark Dataset	Utilizing the Wasserstein Generative Adversarial Network (WGAN), termed Artifact Removal WGAN (AR-WGAN)	Achieves promising performance: correlation coefficient up to 0.726, with low temporal RRMSE of 0.176 and spatial RRMSE of 0.761.	Reduce energy in low-frequency bands and struggles to remove additional noise without distorting the original energy in the 0–20 Hz range.

Abhay B. Nayak et al.^23^ developed an effective approach for eliminating motion artifacts from EEG signals using the empirical wavelet transform (EWT) technique. Initially, the EEG signals are decomposed into narrowband signals known as intrinsic mode functions (IMFs). The first step involves employing principal component analysis (PCA) to suppress noise from the decomposed IMFs. In the second step, they identify IMFs with noisy components using a variance measure and subsequently remove the identified sections. For their experiments, they utilize publicly available EEG data from the Physionet dataset. Their IMF-variance-based method proves to be more efficient than PCA-based approaches.

A discrete wavelet transform-based technique for eliminating artifacts from EEG signals-blink artefacts in particular-is presented by Wenjia Gao et al.^24^. The technique consists of multiple steps: first, intervals containing blink artifacts are detected using a fixed-length window and a forward-backward low-pass filter (FB-LPF). The best representative blink signal is then used to build an adaptive bi-orthogonal wavelet (ABOW). Lastly, discrete wavelet transform-(DWT) and ABOW are used to filter the discovered signals. The DWT’s decomposition depth is automatically determined by comparing the signals that contain artefacts. They use a semi-simulated EEG dataset for their research, in which they capture 200 Hz signals from 54 healthy people without blinking, normalise, scale, and add ten various amplitude blink signals taken from the dataset.

Yuanzhe Dong et al.^25^ proposed an EEG denoising technique called Artefact Removal WGAN (AR-WGAN), which used the Wasserstein Generative Adversarial Network (WGAN). Its efficacy is assessed using a dataset that is made available to the public as well as one that was acquired on its own. The main contribution is providing a WGAN-based end-to-end denoising model that can effectively filter massive amounts of unprocessed EEG data. The generator, which consists of 1D convolutional and transposed convolutional layers, produced fake data, which produced output close to 0, which the discriminator discriminated from genuine data, which was produced around 1. The public EEGdenoiseNet Benchmark Dataset, which comprises semi-synthetic data with clean EEG segments along with segments of ocular and muscular artifacts, is used by the authors for experimental validation. They also use a self-assembled dataset that includes the raw EEG data from four healthy controls.

The literature reviews are detailed in Table 1, describing each work’s dataset, methodologies, performance, and limitations.

## Generative adversarial network

Proposed in 2014^26^, Generative Adversarial Networks (GANs) is a new method for semi-supervised and unsupervised learning. They accomplish this by modeling high-dimensional data distributions informally. The two primary parts of a GAN are the discriminator and the generator. The discriminator contrasts the bogus data produced by the generator with the genuine data. Most importantly, the generator can only learn through interacting with the discriminator; it does not have direct access to actual images. The discriminator can work with samples taken from a stack of real photos as well as generated samples. By identifying if the image originated from the generator or the real stack, the discriminator receives an error signal. This same error signal, through the discriminator, is used to train the generator, guiding it toward producing higher-quality forgeries. Using Equation  1, the cost of training the GAN is calculated.^27^:

1 $$\begin{aligned} \begin{aligned} \min _{Gen} \max _{Dis} F(Dis,Gen)&= {\mathbb {E}}_{x \sim P(X)} [\log Dis(x; \theta _D)] \\&\quad + {\mathbb {E}}_{x \sim P(Z)} [\log (1 - Dis(z; \theta _{\text {Gen}}))] \end{aligned} \end{aligned}$$

Additionally, the loss function of GANs is expressed as:

2 $$\begin{aligned} \begin{aligned} L = \min _{Gen} \max _{Dis} [\log Dis(x) + \log (1 - Dis(Gen(z)))] \end{aligned} \end{aligned}$$

Three guiding concepts form the basis of GAN’s operational framework^28^:

- Enable the learning of the generative model so that data can be generated using random representation.
- Training the model in a conflicting situation where the discriminator and generator are in opposition.
- Training the system as a whole with artificial intelligence algorithms and deep learning neural networks.

Granted that they function well for semi-supervised and reinforcement learning, GAN networks are essentially used for unsupervised machine learning techniques. Together, these elements provide GANs with end-to-end solutions across a range of industries, including finance, healthcare, and mechanical.

## Materials and methods

EEG data is prone to artifacts, and there is currently no algorithm in the literature that can completely eliminate all artifacts. This work seeks to employ GAN as a method of artifact removal covering most artifacts because of its reconstructive approach. This study aims to denoize EEG data for sophisticated Brain-Computer Interface (BCI) analysis. The proposed method entails feeding noisy signals through a generator to generate clean versions, which are then compared to actual clean signals by a discriminator. This iterative process facilitates the effective denoising of EEG signals. Figure 3 illustrates the proposed models architecture, providing insight into the proposed approach to denoising EEG signals.

### Data recording

Building a computational dataset is crucial to training a deep learning model. A novel dataset with nine different artifacts for training and evaluating the model performance was created. These data were gathered from five subjects studying at Gauhati University, Assam, India. The subjects signed written consent for the EEG recording. Subjects performed several tasks while their EEG signals were recorded. These tasks included blinking, eye-movements, chewing, and others. Every participant took part in multiple recording sessions, providing us with different samples for efficient training and testing of the proposed model.

**Fig. 1:**
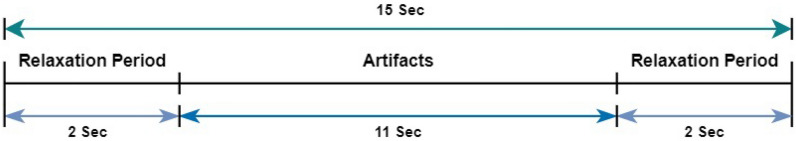
Data recording session paradigm, first and last 2-s is relaxing period.

The data recording session began with a 2-s relaxing period during relaxation the subject was directed to remain motionless and relax. Following the relaxing phase, the subject was guided to blink continuously for 11 s. The process is mentioned in Fig. 1. The primary goal of the given task was to capture the electrical activity that goes along with blinking the eyes. Subsequently, another 2-s relaxation period ensued. Following the previous phase, the process was repeated, this time directing the participant to intentionally move their eyes for 11 s. The motive of the task is to capture electrical signals of the brain related to eye-movement. This was followed by another 2 s relaxation period, allowing the subject to recover and go back to their initial state before moving on to the next phase.

During the data collection session, this pattern is alternating between specific activities and relaxation intervals. Data was gathered for nine different artifacts including eye blinking, eye-movement, chewing, clenching teeth, swallowing movement, tongue movement, jaw movement, head movement, and shoulder movement.

However, during the recording process, subjects naturally blinked their eyes, which introduced an additional artifact. Therefore, the EEG segments contain the main artifact of interest along with the eye blink artifact. This was considered in the artifact removal process.

The data were recorded from thirty two channels using the international 10–20 system for electrode placement, at a sampling frequency of 128 Hz. A wearable EMOTIV-EPOC Flex Gel Kit with thirty two channels was used to collect data. A total of 117 s of data were recorded for each subject, resulting in a total data length of 14,976 samples per subject.

### Data pre-processing

Preprocessing procedures are very essential part for EEG data analysis to improve the data quality and prepare them for more effective analysis. Given that the frequency range of 0.5 to 40 Hz is often where the main characteristics of EEG signals can be discovered, so, the raw EEG data was filtered within this range. This filtering process was effective in removing high-frequency noise, like linear noise at 50 or 60 Hz, while also correcting baseline drift^29^.

Subsequently, the original signals were normalized to limit the signal range. During activities such as eye blinking, facial muscle movement, or other artifacts, the amplitude values of EEG channels can experience sudden high fluctuations. Uncontrolled EEG values have the potential to cause instability in the GAN model because different subjects may show different ranges of uncertainty in their data. To address this issue, min-max normalization is used^30^. This method scales the EEG within a specified range, typically from − 1 to 1, ensuring consistency across all subjects.

After the normalization, each artifact is clipped from the raw normalized signal. This separation makes efficient management and independent analysis of artifacts while preserving their characteristics, temporal patterns, and associated features.

In used dataset, each artifact spans 11 s and is preceded and followed by a 2-s relaxation period, with the final 2 s overlapping with the relaxation period of the subsequent artifact, resulting in a total artifact duration of 15 s.

The EEG was captured from thirty two channels at a 128 Hz sampling rate. To extract all artifacts, the number of samples is calculated by multiplying the sampling rate with the length of the artifact plus the relaxation period (11 + 2 + 2). Mathematically, the total number of frames to be clipped (F) is determined as follows:

3 $$\begin{aligned} F = T \times 128 \end{aligned}$$

Where F represents the total frames to be clipped and T represents the duration of the activity.

**Fig. 2:**
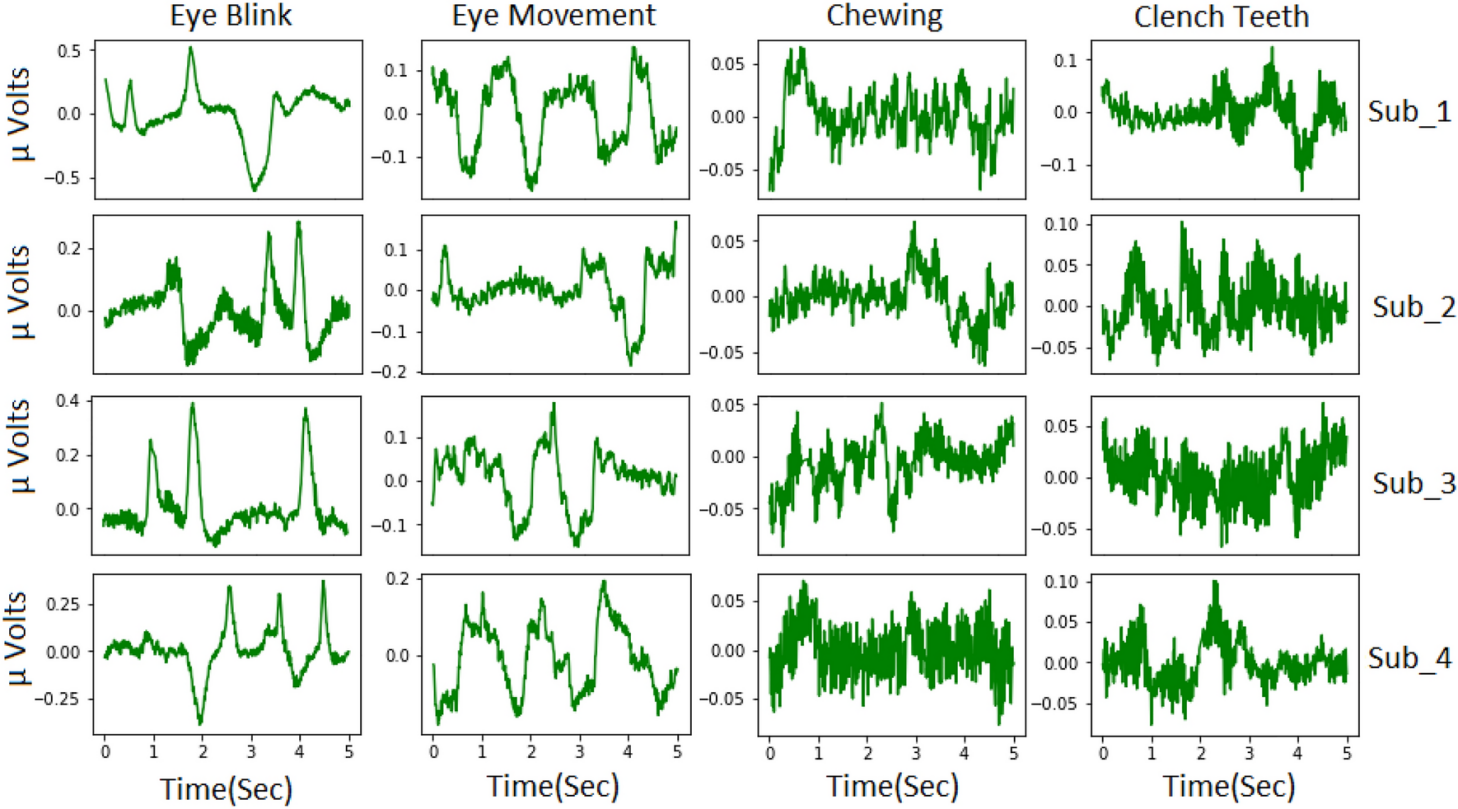
5-s EEG segments of blinking, eye-movements, chewing and clenching teeth from sub1 sub2 sub3 and sub4.

After that, a 5-s window size was adopted to segment and each 15-s artifact was divided into non-overlapping 5-s segments. This results in the extraction of three segments from each channel, totaling 96 segments from all 32 channels. Considering data collection from five subjects, a total of 480 segments are collected. Similarly, 480 segments are collected for each artifact type. Additionally, Fig. 2 illustrates a segment of eye blink, eye-movement, chewing, and clench teeth artifacts from the first four subjects.

The segments are divided into two groups, adopting a ratio of 80:20 for training and testing purposes. Specifically, portions from four subjects were employed for training, and the segments from the fifth subject were saved for testing. A diverse dataset is ensured by this allocation for the model evaluation. Eyeblink, eye-movement, chewing, and clench teeth artifact segments from the four subjects are used for training, while the same artifact segments from the fifth subject are reserved for testing. Overall, 1536 EEG segments are allocated for training, with an additional 384 segments designated for testing.

### Model architecture

**Fig. 3:**
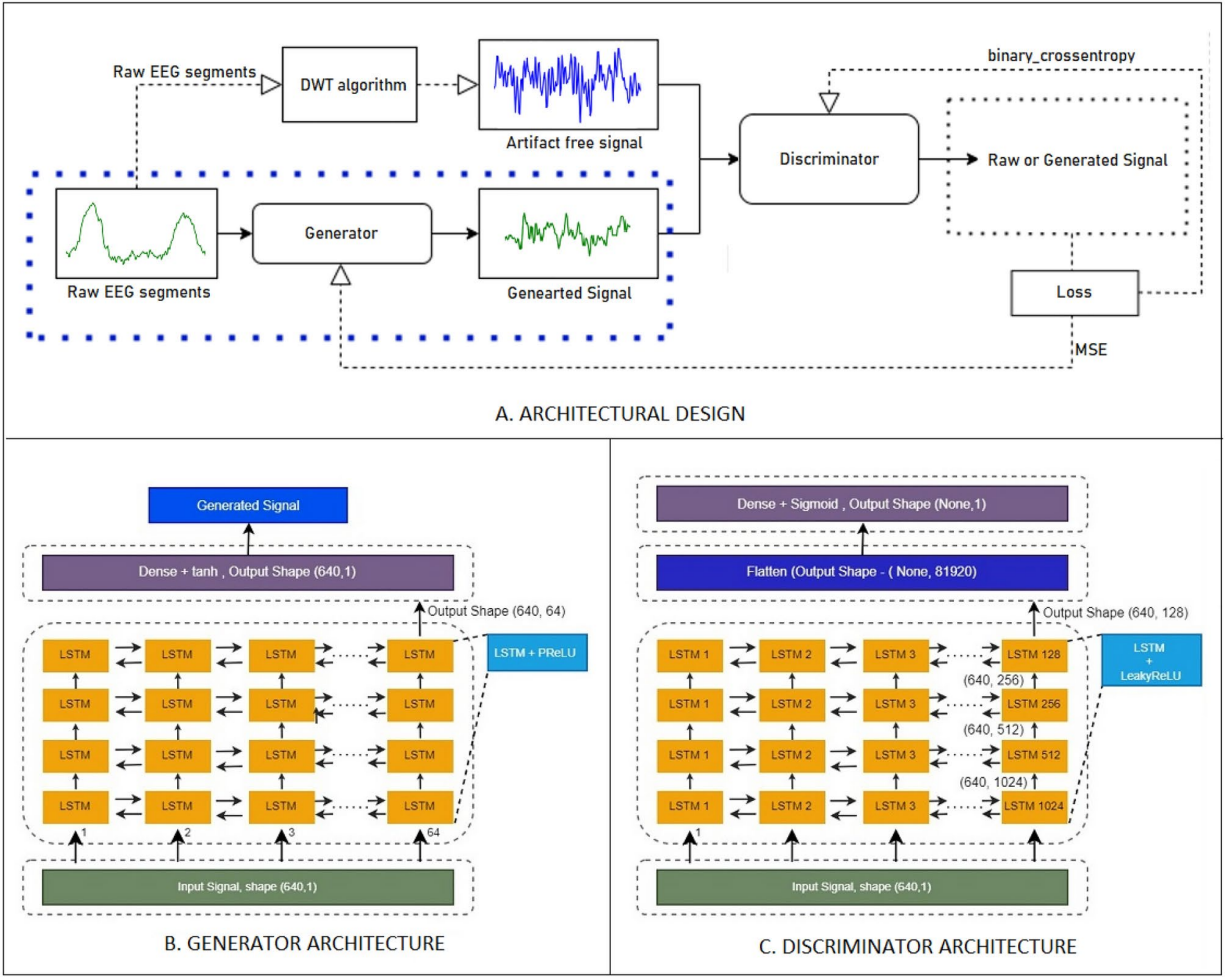
(**A**) Architectural Design of the Model B. Generator Architecture with Layered Model, C. Discriminator Architecture with Layered Model.

The proposed investigation used a recurrent neural network architecture in addition to the Generative Adversarial Network (GAN) concept first presented by Ian Goodfellow^31^. The high-frequency noise, such as linear noise at 50 or 60 Hz, was successfully eliminated by this filtering procedure. As demonstrated in Fig. 3A, the design consists of two primary parts: the discriminator and the generator. Figure 3B,C show the architecture of the generator and the discriminator, respectively.

The generator’s main goal was to generate clean EEG signals from raw input data that had been distorted by things like teeth clenching, eye blinking, and movement. On the other hand, the discriminator receives training to discern between the clean ground truth signals and the produced signals.

From the artifacted EEG signals $S^\text {artifacted}$ in the training dataset, wavelet decomposition (DWT) artifact removal method was applied to generate the clean target EEG signal *S*. These clean signals served as the reference baseline. Subsequently, $S^\text {artifacted}$ was utilized along with their corresponding clean signal *S* as inputs, to train the generator. The focus was to produce artifact-free signal $S'$.

A two-step cleaning technique was employed, in the wavelet denoising method. First, each row of the data matrix was selected for subtracting the average trend from signal using Golay filtering. This initial step aids in eliminating any underlying trends that might obscure the EEG data. Then a thresholding method for the wavelet coefficients of each line was used to solve the noise removal problem. This involved decomposing the signal using the wavelet transform, determining the threshold of detail factors, and correspondingly reconstructing the signal. The thresholding process was determined by the third detail coefficient’s standard deviation, multiplied by a factor of 0.8.

To analyse serial data, the generator model was constructed using LSTM. When data sequences are followed by an activation function called a rectified parametric linear unit (PReLU), it can identify long-term relationships in those sequences. The architecture is described in Table  2. Using both the input and hidden states at each timestep, the LSTM produces the final output in a recurrent feedback loop. To enable the activation function to adaptively learn the rectification parameter for each neuron during training, PReLU introduce learnable parameters to it. This helps to reduce the vanishing gradient problem that deep networks frequently face^32^. With a ’tanh’ activation function, the Dense layer is the last in the model. The dense layer is used to convert the characteristics that the LSTM layers have learned into the final output format. The “tanh” activation function overwrites the output in the range [− 1, 1], which is typically used in GANs to create generator outputs. The generator output is then compared with the real data to calculate the RMSE(root mean square error).

LSTM layers make up the majority of the discriminator’s architecture when it comes to designing discriminator blocks. The LSTM layer is followed by a LeakyReLU activation function with a slightly decline in slope (alpha=0.2). In the LSTM layer, the number of units decreases to 64 from 1028. A Flatten layer is used after the LSTM layer. Next, a single neuron Dense layer is employed. The sigmoid activation function is applied to the Dense layer’s output. Figure 3 describes the model architecture.

### Model training and implementation

In the phase of model training, the generator was fed with training data, from which the generator generated fake samples. These generated data were then sent to the discriminator block, where clean data of the corresponding artifacted segments were already present.

Specifically, 80% of the data was set aside for training, and this data was filtered using the wavelet decomposition technique beforehand. Thus, the generated signal is compared with its respective clean signal. The discriminator loss-function was set to binary cross-entropy, while the generator loss function was set to mean squared error (MSE). Both loss functions were optimized using the Adam optimizer, configured with a learning rate of 1e−3, $\beta _1$ at 0.9, $\beta _2$ at 0.999, and $\epsilon$ at 1e−08.

To prevent the discriminator from being updated during the generator training phase, it is set to non-trainable. The discriminator compares the generated data with the clean data at the end of each epoch to determine the MSE loss. The generator weights are then updated based on this loss. Additionally, the GAN loss was weighted with [1.0, 5e−4], applied to MSE and binary cross-entropy, respectively. This process helped the generator produce a signal $S'$ that closely resembled the ground truth signal *S*. The model was trained with a batch size of 128 over 500 epochs.

**Table 2:** Parameters for the Generator and Discriminator models.

Components	Layers	Hidden units	Input shape	Output shape
Generator	Input		(640, 1)	(640, 1)
	lstm	64	(640, 1)	(640, 64)
	p_relu		(640, 64)	(640, 64)
	lstm_1	64	(640, 64)	(640, 64)
	p_relu_1		(640, 64)	(640, 64)
	lstm_2	64	(640, 64)	(640, 64)
	p_relu_2		(640, 64)	(640, 64)
	lstm_3	64	(640, 64)	(640, 64)
	p_relu_3		(640, 64)	(640, 64)
	dense		(640, 64)	(640, 1)
Discriminator	Input		(640, 1)	(640, 1)
	lstm	1024	(640, 1)	(640, 1024)
	leaky_re_lu		(640, 1024)	(640, 1024)
	lstm_1	512	(640, 1024)	(640, 512)
	leaky_re_lu		(640, 512)	(640, 512)
	lstm_2	256	(640, 512)	(640, 256)
	leaky_re_lu		(640, 256)	(640, 256)
	lstm_3	128	(640, 256)	(640, 128)
	leaky_re_lu		(640, 128)	(640, 128)
	flatten		(640, 128)	(None, 81920)
	dense		(None, 81920)	(None, 1)
	Activation		(None, 1)	(None, 1)

Once the training process is completed, the trained generator and discriminator models are obtained. However, the generator is needed to purify the signal. The discriminator’s main job is to deliver loss, which directs the generator to adjust its weights to produce more precise signals. After successful training, it was required to send the 5-s segmented normalized data, which was stored for testing purposes. Specifically, 20% of the data was reserved for testing. This test data is then fed into the trained generator to produce cleaned EEG signals, enabling us to evaluate the performance of the model on unseen data.

### Evaluation methods

Five metrics are primarily utilised to assess the performance of the signal produced by the suggested model: CC (Correlation Coefficient), RMSE (Root Mean Square Error), NMSE (Normalised Mean Square Error), Signal to Noise Ratio (SNR) and Signal to Artifact Ratio (SAR).

#### Normalized mean squared error (NMSE)

It is a metric, used to confirm whether the generated signal by the model is similar to the actual EEG signal. In addition to visual examination, the Normalised Mean Squared Error (NMSE) measures the average squared difference between the Raw and Generated signals, normalised by the original signal’s variance. Mathematically, it is presented as:

4 $$\begin{aligned} \text {MSE}= & \frac{{\Vert \text {orgSig}(:) - \text {recSig}(:) \Vert }_{2}^2}{\text {length}(\text {orgSig}(:))} \end{aligned}$$

5 $$\begin{aligned} \text {NMSE}= & \frac{{\text {MSE}}}{\Vert \text {orgSig}(:) \Vert _{2}^2} \end{aligned}$$

Here, orgSig(:) represents the original signal, recSig(:) represents the reconstructed signal.

NMSE provides a measurement of the relative error between the original (raw signal) and generated signals, considering the scale of the signals. NMSE values close to zero indicate a good match between the original (raw signal) and generated signals, while higher values indicate larger discrepancies.

#### Root mean squared error (RMSE)

Without normalization, the RMSE offers a metric comparable to the NMSE. It represents the square root of the average squared difference between corresponding samples of the original and reconstructed signals. Mathematically, it is expressed as^33^:

6 $$\begin{aligned} \text {MSE}= & \frac{\sum _{i=1}^{N} (data_i - \text {estimate}_i)^2}{\text {numel}(data)} \end{aligned}$$

7 $$\begin{aligned} \text {RMSE}= & \sqrt{\text {MSE}} \end{aligned}$$

Here, $\text {data}_i$ and $\text {estimate}_i$ represent the $\text {i}$-th elements of the data and estimate vectors, respectively. The total number of elements in the data vectors are defined by N. *numel*(*data*) indicates the number of elements in the *data* vector.

RMSE quantifies the average size of the errors between the generated and original signals. It presents an absolute measure of the error, regardless of the scale of the signals. Lower RMSE (Root-Mean-Square Error) values define better agreement between the original (raw signal) and reconstructed signals.

#### Correlation coefficient (CC)

Correlation Coefficient (CC) is employed to evaluate the model’s capability to remove artifacts while maintaining the brain signal data. It measures how closely relate original (raw signal) and reconstructed signals to each other linearly. Mathematically, CC is expressed as:

8 $$\text {CC} = \frac{{\sum _{i=1}^{N}}({{Sig}_{i}} - {\bar{Sig}}) ({{Sig}^{\prime}_{i}} - {\bar{Sig}^{\prime}})}{\sqrt{{\sum _{i=1}^{N}}({{Sig}_{i}} - {\bar{Sig}})^2}  {\sqrt{{\sum _{i=1}^{N}}({{Sig}^{\prime}_{i}} - {\bar{Sig}^{\prime}})^2}}}$$

Here, *Sig* and $Sig^{\prime}$ represent the $i-$th sample of the original and generated signals, respectively, and ${\bar{Sig}}$ and $Sig^{\prime}$ represent the mean of the original (raw signal) and generated signals. Respectively, Total number of samples is represented by *N*.

How well the variation of one signal predicts the variation of the others is measured by CC. Its values range from − 1 to 1, where 0 denotes no correlation, − 1 represents a perfect negative correlation, and 1 represents a perfect positive correlation. Higher CC values indicate stronger linear agreement between the original (raw signal) and reconstructed signals. Thus, CC helps assess the model’s ability to preserve essential brain signal data while removing artifacts.

#### Signal to noise ratio (SNR)

SNR is employed to evaluate the effectiveness of artifact removal by comparing the signal power within a specific frequency band to the noise power. The formula used for calculating the SNR in decibels (dB) is:

9 $$\begin{aligned} \text {SNR}_{\text {band}} = 10 \times \log _{10}\left( \frac{P_{\text {signal}}}{P_{\text {noise}}}\right) \end{aligned}$$

$P_{\text {signal}}$ is the power of the EEG signal within a particular frequency band, calculated by summing the power spectral density (PSD) values within the frequency band of interest.

10 $$\begin{aligned} P_{\text {signal}} = \sum _{k \in \text {Band}} |Y(k)|^2 \end{aligned}$$

*Y*(*k*) represents the FFT coefficient at the $k^{\text {th}}$ frequency bin.

The power of the noise is then calculated by subtracting the signal power from the total power of the entire signal:

11 $$\begin{aligned} P_{\text {noise}} = \sum _{k=0}^{N-1} |Y(k)|^2 - P_{\text {signal}} \end{aligned}$$

in the equation, $\sum _{k=0}^{N-1} \left| Y(k) \right| ^2$ is the total power of the signal, summed across all frequency.

A higher SNR indicates a cleaner signal with less residual noise, confirming the model’s effectiveness in separating the desired EEG signal from unwanted artifacts^35^. This metric demonstrates how well the artifact removal process enhances the signal quality, ensuring that the EEG data is more representative of the true neural activity, with minimal distortion from external noise sources.

#### Signal to artifact ratio (SAR)

Signal-to-Artifact Ratio (SAR) measures the effectiveness of a methodology by comparing the variance of the artifact signal to the variance of the residual artifact after applying a given methodology. Mathematically, SAR is expressed as:

12 $$\begin{aligned} \text {SAR} = 10 \times \log _{10} \left( \frac{\sigma (A)}{\sigma (A - A')}\right) \end{aligned}$$

where $A$ represents the Artifact signal, and $A'$ represents the cleaned signal. Higher SAR value indicates a more effective artifact removal, suggesting that the resulting signal is cleaner and more representative of the underlying brain activity.

## Results and discussion

Following the completion of all training and testing phases with success, the proposed denoising model demonstrated impressive efficacy in removing noise (Artifacts) from EEG signals while preserving essential brain signal data.

The proposed model underwent rigorous optimization using 80% of the dataset during the training stage. The trend of decreasing loss values over time indicates that the model demonstrated consistent convergence. This implies that during the training stage, the model successfully learned to denoise EEG signals.

Utilizing the remaining 20% of the dataset in the testing phase, the model exhibited robust performance. After that the quantitative assessment was performed using metrics such as Normalized Mean Squared Error-NMSE, Root Mean Squared Error-RMSE, Correlation Coefficient-CC, Signal to Noise Ratio-SNR and Signal to Artifact Ratio-SAR. These metrics provided a comprehensive evaluation of how closely the generated signals matched the original EEG signals, reflecting a strong agreement between them.

**Fig. 4:**
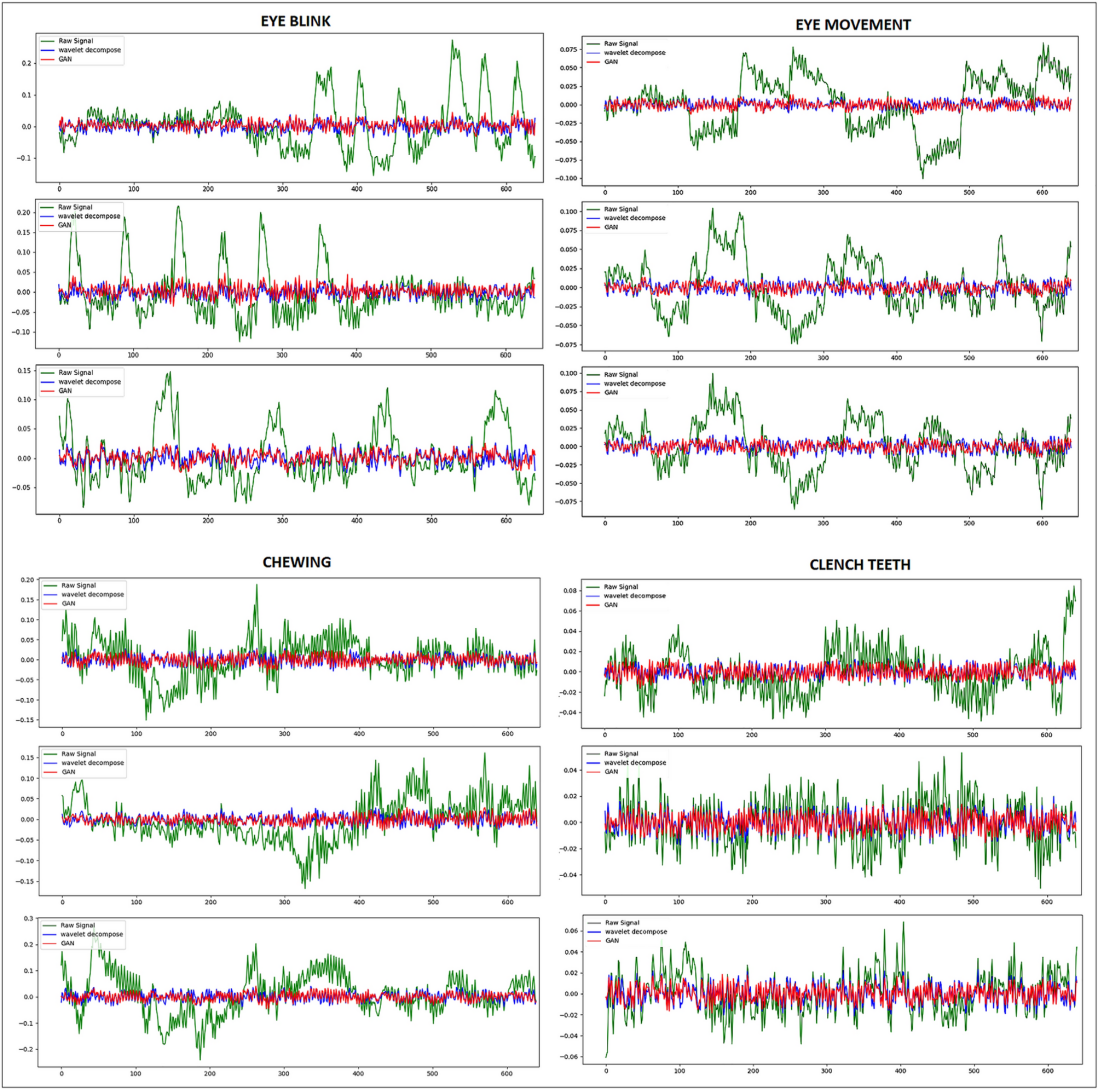
Raw EEG signals (Green), Wavelet Denoised EEG signals (Blue) and generated signal by the proposed model (Red) of Eye Blink and eye-movement, Chewing and Clench Teeth artifacts.

In Fig. 4, segments of each artifact (eye blink, eye-movement, chewing, clench teeth) are presented. The figure exhibits the raw EEG signals alongside those generated by the proposed model and signals cleaned using wavelet decomposition methods.

Distinct characteristics of each artifact are observable: eye blink artifacts are denoted by high sudden spikes in the EEG signals, while eye-movement artifacts display square waves. Chewing artifacts appear as rhythmic and repeated patterns with moderate to high amplitude, and clenching teeth artifacts are typified by high-amplitude spikes.

The raw EEG signal are represented by the green signal, while the signals highlighted in red and blue depict the corrected signals after artifact removal. These denoised signals demonstrating how well the method works to reduce artifacts while preserving the integrity of the EEG data. In smoother waveforms, The removal of artifacts results, facilitating more accurate analysis of underlying brain activity. After denoising, the red signal successfully reduces the abrupt oscillations found in the green signal, as can be seen by comparing the two signals. The comparison clearly illustrates how the proposed method effectively reduces artifacts while maintaining the integrity of the underlying EEG data. The denoised signals provide strong evidence of the model’s capability to preserve essential brain activity while minimizing unwanted noise, thus ensuring a cleaner and more accurate representation of the EEG signals.

**Fig. 5:**
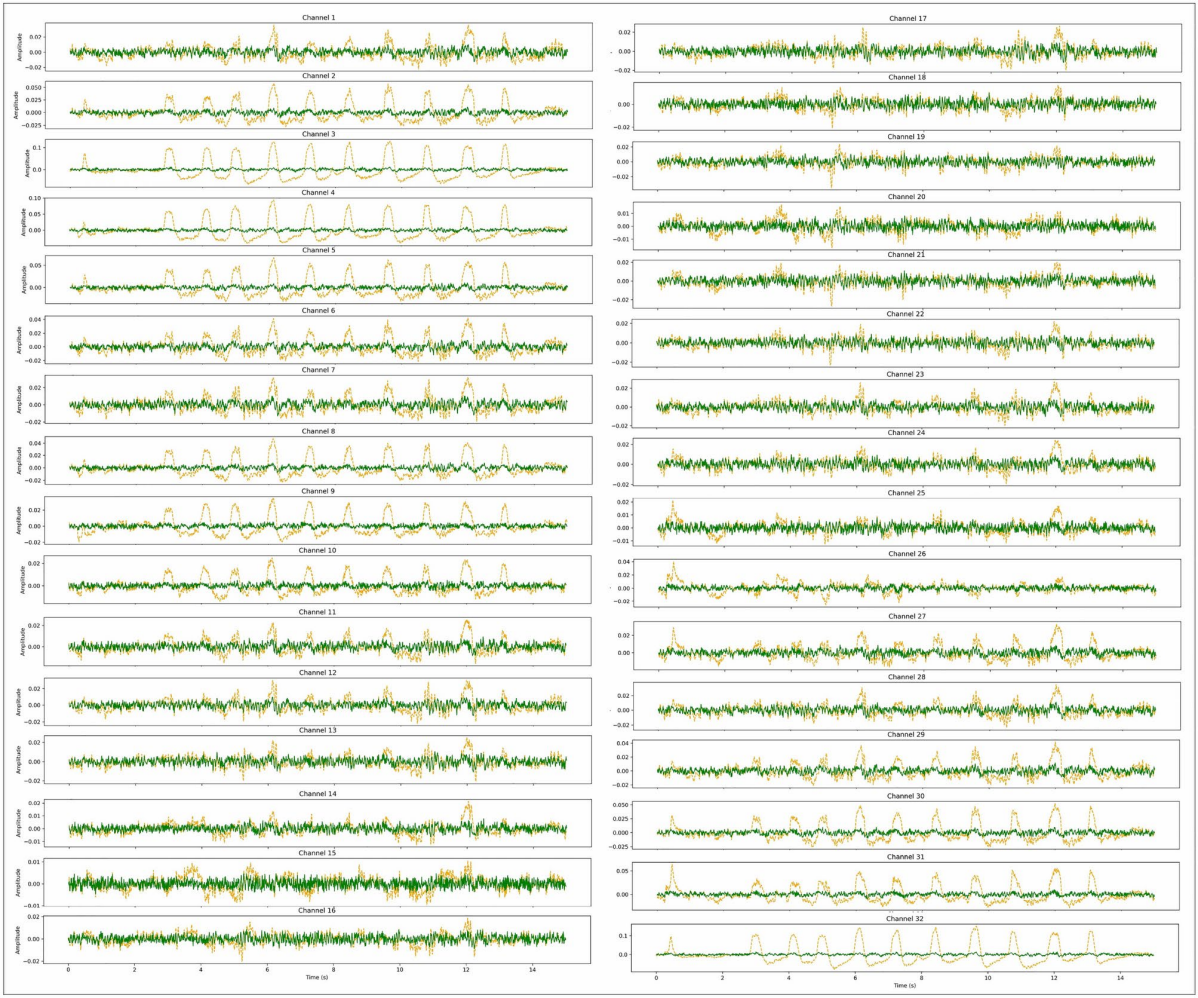
Eye Blink Artifact (Orange) vs. Clean Signal (Green) Across 32 Channels.

In Fig. 5, an EEG signal displaying both the presence of Eye Blink artifact and its clean counterpart across 32 channels is showcased. The clean EEG signal is represented by the green signals, while the artifact affected EEG signal is depicted in orange. This visualization provides a clear comparison between the noisy and clean signals. Additionally, the successful removal of other artifacts, such as eye-movement, chewing, and teeth clenching, from the EEG signals has been documented and made available for review. These results, along with the corresponding EEG images, have been uploaded to the GitHub repository, as referenced in^34^. This allows for further examination and validation of the model’s performance across various types of artifact.

Following the quantitative analysis, which included assessing NMSE, RMSE, CC, SNR and SAR, the generated signals were compared with the original signals, as well as the signals cleaned by wavelet decomposition methods with the original signals. Subsequently, both the GAN-based signals and the wavelet-based signals were compared to determine which performed better.

For examining the characteristics of the signals, RMSE was used, which gives information about the average magnitude of the errors between the generated or wavelet-decomposed signals and the original signals. By focusing on the deviations in signal reconstruction, RMSE helps quantify how closely the denoised signals resemble the true, artifact-free signals.

**Table 3:** The effectiveness of the proposed method for eliminating artifacts using wavelet methods.

RMSE								
	Eye Blink		Eye-movement		Chewing		Clench teeth	
	Wavelet	AnEEG	Wavelet	AnEEG	Wavelet	AnEEG	Wavelet	AnEEG
1	0.0546	**0**.**0545**	0.0319	**0**.**0317**	0.0394	**0**.**0390**	0.0203	**0**.**0201**
2	0.1168	**0**.**1154**	0.0351	**0**.**0347**	0.0626	**0**.**0618**	**0**.**0427**	0.0429
3	0.3113	**0**.**3083**	0.0767	**0**.**0763**	0.1503	**0**.**1488**	0.1579	**0**.**1573**
4	0.2033	**0**.**2014**	0.0349	**0**.**0343**	0.0771	**0**.**0764**	0.0579	**0**.**0577**
5	0.1375	**0**.**1361**	0.0279	**0**.**0274**	0.0737	**0**.**0731**	0.0464	**0**.**0463**
6	0.0749	**0**.**0741**	0.0285	**0**.**0282**	0.0659	**0**.**0649**	**0**.**0282**	**0**.**0282**
7	0.0551	**0**.**0547**	0.0216	**0**.**0213**	0.0614	**0**.**0599**	**0**.**0210**	**0**.**0210**
8	0.0943	**0**.**0934**	0.0206	**0**.**0202**	0.1022	**0**.**1020**	0.0237	**0**.**0232**
9	0.0748	**0**.**0746**	0.0296	**0**.**0290**	**0**.**0525**	0.0527	0.0237	**0**.**0229**
10	0.0475	**0**.**0473**	0.0205	**0**.**0197**	0.0744	**0**.**0737**	0.0184	**0**.**0178**
11	0.0401	**0**.**0395**	0.0143	**0**.**0138**	0.0554	**0**.**0539**	0.0149	**0**.**0146**
12	**0**.**0452**	0.0453	0.0262	**0**.**0260**	**0**.**0484**	0.0496	**0**.**0206**	0.0208
13	**0**.**0335**	0.0339	0.0248	**0**.**0245**	0.0495	**0**.**0482**	0.0206	**0**.**0204**
14	0.0256	**0**.**0254**	0.0126	**0**.**0121**	0.0316	**0**.**0311**	0.0141	**0**.**0135**
15	0.0189	**0**.**0186**	0.0200	**0**.**0195**	0.0317	**0**.**0310**	0.0165	**0**.**0160**
16	0.0255	**0**.**0251**	0.0232	**0**.**0227**	0.0373	**0**.**0361**	0.0168	**0**.**0161**
17	**0**.**0335**	0.0345	0.0299	**0**.**0297**	0.0864	**0**.**0852**	0.0209	**0**.**0211**
18	**0**.**0253**	0.0258	0.0249	**0**.**0245**	0.0352	**0**.**0347**	0.0179	**0**.**0175**
19	**0**.**0332**	0.0343	0.0270	**0**.**0267**	0.0362	**0**.**0356**	0.0190	**0**.**0189**
20	0.0263	**0**.**0261**	0.0278	**0**.**0273**	0.0329	**0**.**0322**	0.0192	**0**.**0188**
21	0.0296	**0**.**0288**	**0**.**0322**	0.0320	0.0374	**0**.**0370**	0.0187	**0**.**0182**
22	0.0286	**0**.**0277**	0.0324	**0**.**0322**	**0**.**0273**	0.0274	0.0149	**0**.**0148**
23	**0**.**0359**	0.0361	0.0318	**0**.**0316**	0.0298	**0**.**0295**	**0**.**0215**	**0**.**0215**
24	**0**.**0322**	0.0322	0.0241	**0**.**0238**	0.0499	**0**.**0493**	0.0185	**0**.**0183**
25	0.0261	**0**.**0260**	0.0195	**0**.**0191**	0.0321	**0**.**0316**	0.0267	**0**.**0265**
26	0.0452	**0**.**0451**	0.0851	**0**.**0848**	0.0854	**0**.**0851**	0.0442	**0**.**0441**
27	0.0538	**0**.**0534**	0.0472	**0**.**0471**	0.0512	**0**.**0511**	0.0382	**0**.**0381**
28	0.0480	**0**.**0479**	0.0373	**0**.**0372**	**0**.**0452**	0.0455	0.0235	**0**.**0221**
29	0.0675	**0**.**0670**	**0**.**0359**	0.0357	0.0508	**0**.**0505**	**0**.**0312**	0.0313
30	0.1032	**0**.**1020**	0.0473	**0**.**0471**	**0**.**0668**	**0**.**0668**	**0**.**0522**	0.0523
31	0.1165	**0**.**1153**	0.0955	**0**.**0950**	**0**.**1790**	0.1799	0.0941	**0**.**0940**
32	0.3474	**0**.**3440**	0.0823	**0**.**0816**	0.1295	**0**.**1285**	0.1303	**0**.**1294**
Mean	0.0753	**0**.**0739**	0.0353	**0**.**0336**	0.0621	**0**.**0609**	0.0355	**0**.**0345**

Values in bold depict better performance.

In Table 3, the RMSE value for each electrode was calculated, comparing the original signal with the wavelet-based signal and the original signal with the signal generated by the model. The RMSE values for the generated signals are consistently smaller than wavelet denoising signals for most electrodes, there are exceptions observed, indicating the superior performance of the proposed model in preserving the integrity of the EEG signals. The average RMSE for the generated eye blink, eye-movement, chewing, and clench teeth signals is **0.0739, 0.0336, 0.0609,** and **0**.**0345** respectively, while the average RMSE for the wavelet-based signal is **0.0753, 0.0353, 0.0621** and **0**.**0355** respectively. It means that the proposed model-generated signal generally showed more effective agreement with the original signals compared to the wavelet-based signals. Lower RMSE (Root-mean-square Error) values indicate better agreement between the original (Raw Signal) and reconstructed signal. Therefore, in conclusion, the signals generated by the proposed model possess more accurate characteristics compared to those obtained through wavelet methods, although some electrodes may require further investigation.

**Table 4:** Performance of the proposed method with respect to wavelet methods in removing artifacts.

NMSE								
	Eye Blink		Eye-movement		Chewing		Clench teeth	
	Wavelet	AnEEG	Wavelet	AnEEG	Wavelet	AnEEG	Wavelet	AnEEG
1	0.0098	**0**.**0072**	0.0160	**0**.**0126**	0.0072	**0**.**0062**	0.0043	**0**.**0036**
2	0.0495	**0**.**0371**	0.0221	**0**.**0164**	0.0121	**0**.**0099**	0.0124	**0**.**0080**
3	0.2305	**0**.**1958**	0.0732	**0**.**0424**	0.0600	**0**.**0378**	0.1038	**0**.**0719**
4	0.1635	**0**.**1244**	0.0224	**0**.**0207**	0.0262	**0**.**0168**	0.0232	**0**.**0156**
5	0.0693	**0**.**0498**	0.0149	**0**.**0120**	0.0163	**0**.**0133**	0.0156	**0**.**0108**
6	0.0185	**0**.**0147**	0.0138	**0**.**0109**	0.0126	**0**.**0122**	0.0060	**0**.**0045**
7	0.0104	**0**.**0082**	0.0086	**0**.**0081**	**0**.**0094**	0.0096	0.0032	**0**.**0025**
8	0.0509	**0**.**0412**	0.0080	**0**.**0079**	0.0601	**0**.**0396**	0.0076	**0**.**0075**
9	0.0632	**0**.**0439**	**0**.**0182**	0.0234	0.0222	**0**.**0185**	0.0074	**0**.**0083**
10	0.0312	**0**.**0237**	**0**.**0031**	0.0038	0.0186	**0**.**0154**	0.0037	**0**.**0039**
11	0.0088	**0**.**0075**	**0**.**0053**	0.0071	**0**.**0113**	0.0138	0.0025	**0**.**0023**
12	**0**.**0051**	0.0071	0.0120	**0**.**0098**	**0**.**0068**	**0**.**0068**	0.0028	**0**.**0021**
13	0.0047	**0**.**0033**	0.0107	**0**.**0089**	**0**.**0088**	0.0101	0.0044	**0**.**0035**
14	0.0056	**0**.**0046**	**0**.**0043**	0.0061	0.0051	**0**.**0049**	**0**.**0021**	0.0023
15	0.0052	**0**.**0046**	0.0074	**0**.**0078**	0.0056	**0**.**0050**	**0**.**0025**	0.0026
16	0.0034	**0**.**0024**	0.0102	**0**.**0083**	**0**.**0041**	0.0042	**0**.**0018**	0.0019
17	0.0044	**0**.**0028**	0.0145	**0**.**0106**	0.0217	**0**.**0197**	0.0024	**0**.**0018**
18	0.0036	**0**.**0024**	0.0119	**0**.**0100**	0.0041	**0**.**0036**	0.0019	**0**.**0017**
19	0.0037	**0**.**0020**	0.0129	**0**.**0099**	0.0047	**0**.**0040**	0.0020	**0**.**0017**
20	0.0048	**0**.**0033**	0.0150	**0**.**0126**	0.0065	**0**.**0058**	0.0035	**0**.**0033**
21	0.0035	**0**.**0020**	0.0184	**0**.**0145**	0.0080	**0**.**0071**	0.0037	**0**.**0035**
22	0.0034	**0**.**0021**	0.0182	**0**.**0139**	0.0042	**0**.**0034**	0.0021	**0**.**0016**
23	0.0056	**0**.**0039**	0.0171	**0**.**0128**	0.0051	**0**.**0046**	0.0032	**0**.**0025**
24	0.0047	**0**.**0032**	**0**.**0159**	0.0161	0.0123	**0**.**0119**	0.0038	**0**.**0030**
25	0.0054	**0**.**0044**	**0**.**0126**	0.0165	0.0046	**0**.**0040**	0.0059	**0**.**0047**
26	0.0121	**0**.**0085**	0.1011	**0**.**0700**	0.0347	**0**.**0242**	0.0156	**0**.**0114**
27	0.0146	**0**.**0121**	0.0452	**0**.**0356**	0.0107	**0**.**0076**	0.0108	**0**.**0074**
28	**0**.**0053**	0.0079	0.0244	**0**.**0187**	0.0087	**0**.**0067**	0.0047	**0**.**0036**
29	0.0156	**0**.**0120**	0.0217	**0**.**0156**	0.0087	**0**.**0074**	0.0069	**0**.**0050**
30	0.0441	**0**.**0333**	0.0386	**0**.**0283**	0.0143	**0**.**0100**	0.0169	**0**.**0108**
31	0.1153	**0**.**1165**	0.1181	**0**.**0805**	0.0811	**0**.**0466**	0.0423	**0**.**0252**
32	0.2605	**0**.**2360**	0.0865	**0**.**0514**	0.0446	**0**.**0287**	0.0689	**0**.**0471**
Mean	0.0388	**0**.**0320**	0.0257	**0**.**0195**	0.0175	**0**.**0131**	0.0124	**0**.**0089**

Values in bold depict better performance.

After assessing the RMSE values, the similarity between the original and noise-free signals using NMSE was evaluated. By calculating NMSE, the model’s ability to accurately reconstruct the clean signal from the noisy input is assessed.

For NMSE, the original signal and the wavelet denoised signal as well as the NMSE value between the original signal and the reconstructed signal were computed. It can be observed that the NMSE values of the generated signals are close to zero relative to the wavelet-denoised signals. Table  4 presents all the calculated NMSE values. For all 32 electrodes, the NMSE values were calculated and averaged, it was found that the average NMSE value for the generated signal by the proposed model for eye blink, eye-movement, chewing, and clench teeth is **0.0320, 0.0195, 0.0131,** and **0**.**0089** respectively, while the average NMSE value for the wavelet-based signals is **0.0388, 0.0257, 0.0175** and **0**.**0124** respectively. This indicates that the proposed model created signal are more similar to the original signal compared to the wavelet-based denoised signals. NMSE values close to zero imply a good match between the original and reconstructed signal, while the higher values of NMSE signify greater discrepancies between the original and reconstructed signal.

**Table 5:** Efficiency of the proposed method in comparison with wavelet methods when storing EEG data.

CC								
	Eye Blink		Eye-movement		Chewing		Clench teeth	
	Wavelet	AnEEG	Wavelet	AnEEG	Wavelet	AnEEG	Wavelet	AnEEG
1	0.6481	**0**.**6861**	0.5775	**0**.**6377**	0.6346	**0**.**6855**	0.5973	**0**.**6486**
2	0.4549	**0**.**5944**	0.5298	**0**.**6220**	0.5431	**0**.**6259**	0.4609	**0**.**5014**
3	0.3654	**0**.**5788**	0.4500	**0**.**5670**	0.3673	**0**.**5169**	0.2246	**0**.**3096**
4	0.2864	**0**.**3757**	0.5240	**0**.**6390**	0.4403	**0**.**5477**	**0**.**4426**	0.3727
5	0.4347	**0**.**5787**	0.5780	**0**.**6802**	0.4951	**0**.**5642**	0.4268	**0**.**4823**
6	0.5508	**0**.**6287**	0.5914	**0**.**6714**	0.5224	**0**.**6039**	0.5663	**0**.**6088**
7	0.4256	**0**.**4864**	0.6553	**0**.**7274**	0.5566	**0**.**6619**	0.6507	**0**.**6928**
8	0.4512	**0**.**5653**	0.6584	**0**.**7447**	0.3470	**0**.**4064**	0.4832	**0**.**5680**
9	0.4433	**0**.**5033**	0.5161	**0**.**6228**	0.4768	**0**.**4823**	0.4647	**0**.**5706**
10	0.4814	**0**.**5491**	**0**.**9432**	0.8768	0.4845	**0**.**5575**	0.6153	**0**.**6918**
11	0.6271	**0**.**7035**	0.7004	**0**.**7719**	0.5042	**0**.**6137**	0.6992	**0**.**7497**
12	0.7085	**0**.**7448**	0.6253	**0**.**6808**	0.6207	**0**.**7091**	0.6964	**0**.**6711**
13	0.7807	**0**.**7992**	0.6289	**0**.**6920**	0.5525	**0**.**6447**	0.5557	**0**.**6192**
14	0.7178	**0**.**7708**	0.7062	**0**.**7908**	**0**.**7716**	0.7139	0.7195	**0**.**7854**
15	0.7183	**0**.**7803**	0.6425	**0**.**7459**	**0**.**7692**	0.6861	0.6947	**0**.**7594**
16	0.8145	**0**.**8288**	0.5966	**0**.**6952**	**0**.**7891**	0.7020	0.7674	**0**.**8340**
17	**0**.**8109**	0.8101	0.5879	**0**.**6558**	0.3987	**0**.**4954**	0.6819	**0**.**7083**
18	0.8116	**0**.**8241**	0.5839	**0**.**6716**	0.7215	**0**.**7876**	0.7506	**0**.**8010**
19	0.8478	**0**.**8636**	0.5761	**0**.**6572**	0.7012	**0**.**7803**	**0**.**7746**	0.7330
20	0.7541	**0**.**7747**	0.5412	**0**.**6384**	0.6466	**0**.**7328**	**0**.**6811**	0.6157
21	0.8352	**0**.**8530**	0.5361	**0**.**6035**	0.6071	**0**.**6678**	**0**.**6695**	0.6020
22	0.8536	**0**.**8599**	0.5420	**0**.**6081**	0.7501	**0**.**7799**	0.7154	**0**.**7586**
23	0.4344	**0**.**4805**	0.5617	**0**.**6219**	0.7100	**0**.**7638**	0.6452	**0**.**6860**
24	0.7616	**0**.**8065**	0.5313	**0**.**6074**	0.5251	**0**.**5826**	0.6130	**0**.**6793**
25	0.7218	**0**.**7596**	0.5409	**0**.**6180**	0.7244	**0**.**7861**	0.5539	**0**.**6117**
26	0.6372	**0**.**6923**	0.3812	**0**.**4463**	0.4330	**0**.**4995**	0.4290	**0**.**4741**
27	0.5706	**0**.**6271**	0.4480	**0**.**4811**	0.5795	**0**.**6312**	0.4740	**0**.**5290**
28	0.6815	**0**.**7163**	0.5203	**0**.**5640**	0.6138	**0**.**6282**	0.5911	**0**.**6408**
29	0.1702	**0**.**2395**	0.5446	**0**.**6034**	0.6031	**0**.**6539**	0.5446	**0**.**5868**
30	0.4678	**0**.**5957**	0.4723	**0**.**5346**	0.5450	**0**.**5952**	0.4186	**0**.**4669**
31	0.4601	**0**.**5958**	0.3866	**0**.**4844**	**0**.**3677**	0.3665	0.3253	**0**.**3870**
32	0.3581	**0**.**5802**	0.4307	**0**.**5805**	0.3926	**0**.**5098**	0.2585	**0**.**3753**
Mean	0.6280	**0**.**6985**	0.5638	**0**.**6440**	0.5686	**0**.**6244**	0.5685	**0**.**6100**

Values in bold depict better performance.

These results demonstrates that, in terms of maintaining the similarity between the generated and original noise-free signals, the proposed model performed better than the wavelet-based methods, as seen by the generated signals’ lower average NMSE values.

After the NMSE evaluation, the model was assessed based on the likeness between the original and denoised signals by calculating the correlation coefficient (CC). CC was calculated both between the original signal and the reconstructed signal, as well as between the original signal and the wavelet-denoised signal. All calculated values for the 32 channels are shown in Table 5. The average CC for the generated signal is **0.6985, 0.6440, 0.6244**, and **0**.**61** for eye blink, eye-movement, chewing, and clenching teeth signals, respectively, indicating a strong linear agreement with the original signals. In contrast, the average CC for the wavelet-based signals was **0.6280, 0.5638, 0.5686,** and **0**.**5685** respectively.

A higher CC value indicates a more robust linear relationship between the original and reconstructed signal. In contrast to the wavelet-based denoised signals, the higher average CC value found for the created signals indicates a tighter alignment with the original signal. This indicates the strength of the model, in maintaining the linear relationship between the original and generated signal, confirming its efficiency over the wavelet-based method.

To evaluate the effectiveness of the proposed model, Signal-to-Noise Ratio-SNR was also evaluated. SNR measures the strength of the signal relative to the level of noise, making it an essential indicator of how well the noise has been subtracted and how much the signal power has been enhanced in the generated signals by the proposed model. The SNR across all 32 electrodes for all artifacts: Eye Blink, eye-movement, Chewing, and Clench Teeth was calculated. The signals were divided into four frequency bands: Theta (4–8 Hz), Alpha (8–13 Hz), Beta (13–30 Hz), and Gamma (30–50 Hz).

**Table 6:** Signal to Noise Ratio Comparison for Eye Blink in Different Bands.

Eye Blink				
	Theta	Alpha	Beta	Gamma
RAW Signal	3.24	3.91	2.23	$-$2.95
DWT	6.72	10.26	10.62	**10**.**63**
**Proposed Method (AnEEG)**	**7**.**71**	**10**.**62**	**13**.**08**	10.42

Values in bold depict better performance.

**Table 7:** Signal to Noise Ratio Comparison for eye-movement in Different Bands.

Eye-movement				
	Theta	Alpha	Beta	Gamma
RAW Signal	3.61	$-$0.44	2.69	0.54
DWT	6.20	5.94	12.25	12.98
**Proposed Method (AnEEG)**	**7**.**10**	**6**.**64**	**13**.**54**	**13**.**57**

Values in bold depict better performance.

**Table 8:** Signal to Noise Ratio Comparison for Chewing in Different Bands.

Chewing				
	Theta	Alpha	Beta	Gamma
RAW Signal	3.16	0.59	5.37	5.50
DWT	3.09	5.05	11.69	14.37
**Proposed Method (AnEEG)**	**5**.**51**	**6**.**48**	**12**.**60**	**14**.**73**

Values in bold depict better performance.

**Table 9:** Signal to Noise Ratio Comparison for Clench Teeth in Different Bands.

Clench Teeth				
	Theta	Alpha	Beta	Gamma
RAW Signal	5.09	3.61	7.08	5.73
DWT	3.64	6.16	12.16	13.77
**Proposed Method (AnEEG)**	**5**.**51**	**7**.**27**	**13**.**01**	**14**.**55**

Values in bold depict better performance.

For the Eye Blink artifact(refer Table 6), the proposed model demonstrated superior performance with average SNR values of **7**.**71**, **10**.**62**, **13**.**08**, and 10.42 for the Theta, Alpha, Beta, and Gamma bands, respectively. In comparison, the raw artifacted signal yielded lower SNR values of 3.24, 3.91, 2.23, and $-$2.95. The wavelet-based (DWT) denoised signal showed improvements with SNR values of 6.72, 10.26, 10.62, and 10.63.

For the eye-movement artifact (refer Table 7), the proposed model again outperformed the raw and DWT-cleaned signals. The generated signal achieved average SNR values of **7**.**10**, **6**.**64**, **13**.**54**, and **13**.**57** across the Theta, Alpha, Beta, and Gamma bands, respectively. In contrast, the raw artifacted signal had much lower SNR values of 3.61, $-$0.44, 2.69, and 0.54, while the DWT-cleaned signal reached 6.20, 5.94, 12.25, and 12.98.

For the Chewing artifact (refer Table 8), the proposed model consistently outperformed both the raw and DWT methods. The average SNR values for the generated signal were **5**.**51**, **6**.**48**, **12**.**60**, and **14**.**73** in the Theta, Alpha, Beta, and Gamma bands, respectively. The raw artifacted signal had lower SNR values of 3.16, 0.59, 5.37, and 5.50, while the DWT-cleaned signal yielded 3.09, 5.05, 11.69, and 14.37.

Lastly, for the Clench Teeth artifact (refer Table 9), the proposed model showed superior performance with SNR values of **5**.**51**, **7**.**27**, **13**.**01**, and **14**.**55** in the Theta, Alpha, Beta, and Gamma bands, respectively. The raw artifacted signal exhibited SNR values of 5.09, 3.61, 7.08, and 5.73, while the DWT method resulted in 3.64, 6.61, 12.16, and 13.77.

**Fig. 6:**
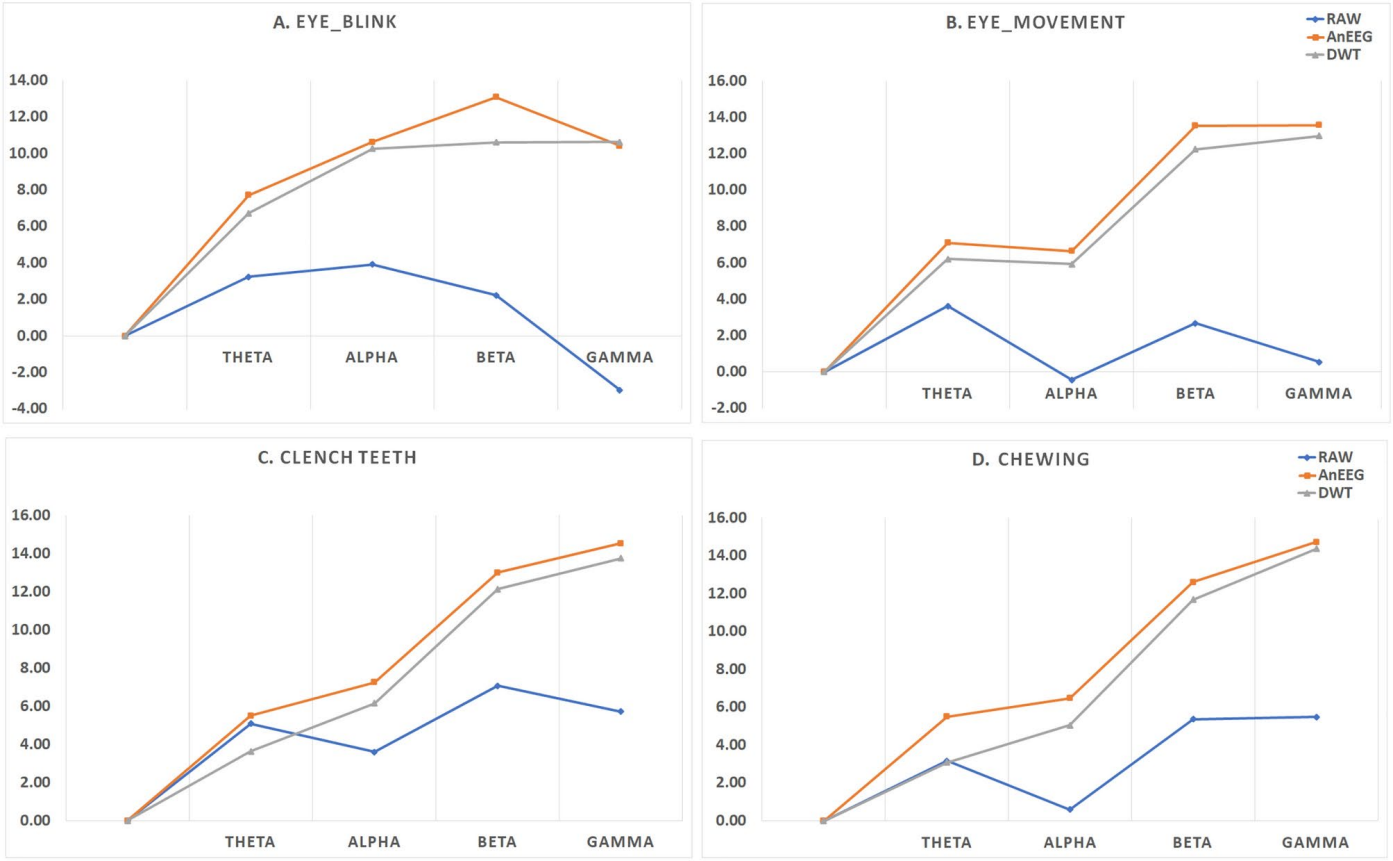
Comparative SNR Analysis Across Frequency Bands for (**A**) Eye Blink, (**B**) Eye-movement, (**C**) Chewing, (**D**) Clench Teeth Using the Proposed Method—AnEEG (Orange), DWT based Method (Grey), and Raw Signals (Blue).

Overall, the results demonstrate that the proposed model consistently achieves higher SNR values across all frequency bands compared to both the raw and DWT-cleaned signals. There is a general trend where the SNR increases with frequency, indicating that the signal quality improves relative to the noise as frequency increases. This suggests that the higher frequency components of the signal are more distinct and less affected by noise in the proposed model compared to the DWT approach. All the values are plotted separately for each artifact for better comparison: the Eye Blink plot is shown in Fig. 6A, the eye-movement plot in Fig. 6B, the Chewing plot in Fig. 6C, and the Clench Teeth plot in Fig. 6D. In these plots, the orange represents the GAN-based signal, the grey represents the DWT-based signal, and the blue represents the raw signal.

After evaluating the Signal-to-Noise Ratio (SNR), we also calculate the Signal-to-Artifact Ratio (SAR) for each artifact. SAR is another important metric that quantifies the ratio between the clean signal and the residual artifact.SAR was calculated for all 32 electrodes across all four artifacts: Eye Blink, Eye-movement, Chewing, and Clench Teeth. Afterward, the SAR values were averaged across all channels to obtain an overall performance measure. The evaluated data is presented in Table 10, showcasing the model’s ability to reduce artifacts.

**Table 10:** Average SAR Values Across 32 Electrodes for Eye Blink, Eye-Movement, Chewing, Clench Teeth Comparing the Proposed Method and DWT based Methods.

SAR								
	Eye Blink		Eye-movement		Chewing		Clench teeth	
	Wavelet	AnEEG	Wavelet	AnEEG	Wavelet	AnEEG	Wavelet	AnEEG
Mean	0.6481	**0**.**6861**	0.5775	**0**.**6377**	0.6346	**0**.**6855**	0.5973	**0**.**6486**

Values in bold depict better performance.

The average SAR value for Eye Blink is ${\textbf {0}}.{\textbf {87}}$ for the GAN-based method, compared to 0.83 for the DWT-based method. For Eye-movement, the GAN approach achieved a SAR of ${\textbf {0}}.{\textbf {62}}$, while DWT achieved 0.50. In the case of Chewing artifacts, the GAN method resulted in a SAR of ${\textbf {0}}.{\textbf {90}}$, while DWT yielded 0.80. Lastly, for Clench Teeth artifacts, the GAN method outperformed DWT with a SAR of ${\textbf {1}}.{\textbf {47}}$ compared to 1.38. In all artifact, the proposed method demonstrated superior performance in artifact removal compared to DWT.

After testing and evaluating all the criteria on the split artifact dataset, the model was also analysed on an additional dataset, “SAM-40”^36^ which was recorded at Gauhati University, Department of Information Technology, Guwahati, Assam , India. This dataset was gathered from 40 subjects to monitor induced stress while performing tasks such as the Stroop color-word test, arithmetic tasks, and mirror image recognition tasks.

The same pre-processing technique was used on this dataset like the proposed dataset. Then, taking one subject’s data to clean artifact, and then analyzed the RMSE, NMSE, CC, SNR, and SAR for the clean signals generated by the proposed model. The RMSE, NMSE, and CC values were calculated for all 32 electrodes and then averaged. These values are described in Table 11. Additionally, SNR and SAR values are presented in Table 12.

**Table 11:** Average RMSE, NMSE, and CC Values for All 32 Electrodes on the SAM-40 Dataset Comparing the proposed Methods and DWT-based Methods.

	RMSE		NMSE		CC	
	Wavelet	AnEEG	Wavelet	AnEEG	Wavelet	AnEEG
Mean	0.0831	**0**.**0819**	0.0164	**0**.**0066**	0.5273	**0**.**5894**

Values in bold depict better performance.

**Table 12:** Average SNR Values Across Frequency Bands and SAR on the SAM-40 Dataset.

	Signal to Noise Ratio (SNR)				
	Theta	Alpha	Beta	Gamma	SAR
RAW Signal	2.32	2.90	4.33	0.63	–
DWT	5.31	8.62	11.79	11.43	1.09
**Proposed Method (AnEEG)**	**6**.**95**	**9**.**72**	**13**.**4**	**12**.**6**	**1**.**34**

Values in bold depict better performance.

In the SAM-40 dataset, the RMSE for the proposed model’s generated signal is **0**.**0819**, compared to 0.0831 for DWT. The NMSE for the generated signal is **0**.**0066**, while DWT’s NMSE is 0.0164. The CC for the proposed model’s generated signal is **0**.**5894**, compared to 0.5273 for DWT. These results indicate that, once again, the proposed model outperforms DWT, with lower RMSE and NMSE values, and a higher CC value.

**Fig. 7:**
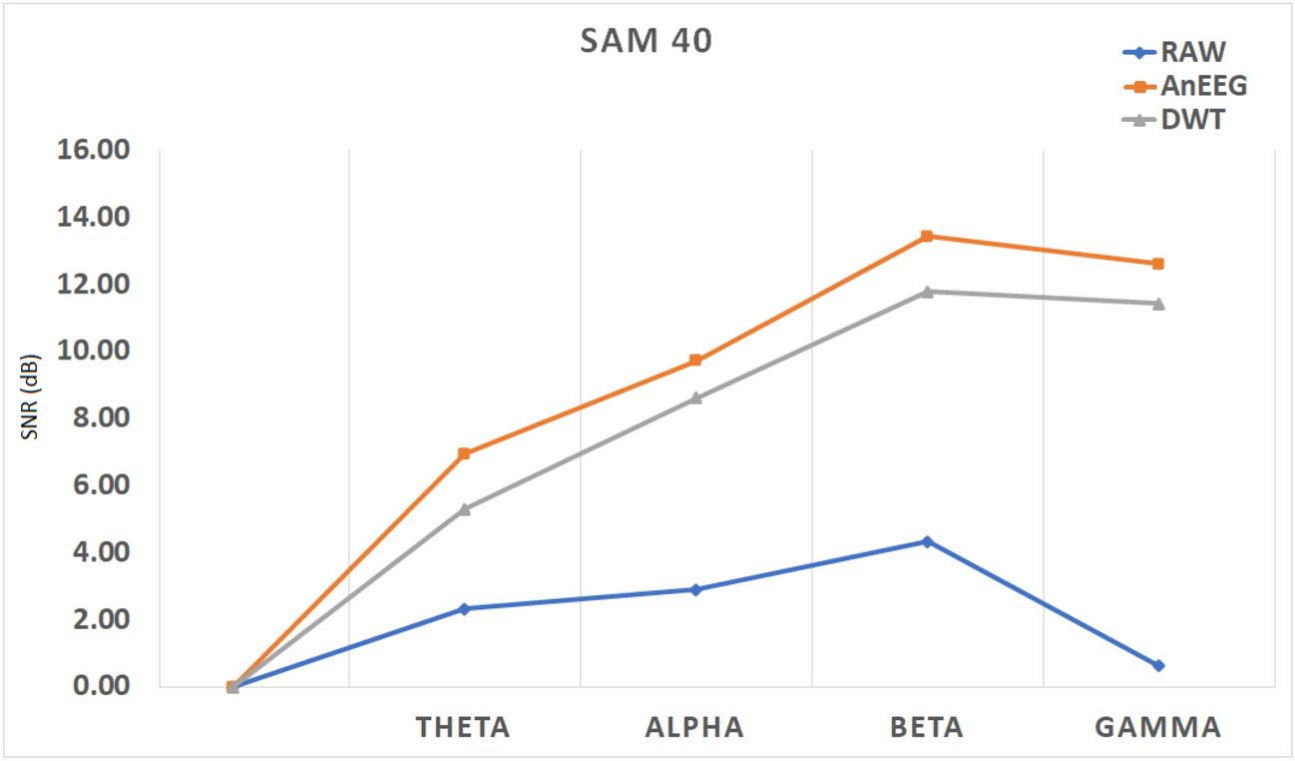
Comparative SNR Analysis Across Frequency Bands for “SAM-40” Dataset Using the Proposed Method (AnEEG) (Orange), DWT based Method (Grey), and Raw Signals (Blue).

The SNR and SAR values are preseneted in Table 11, the SNR was calculated across different frequency bands. The SNR for the proposed model’s generated signal is **6**.**95** in the Theta band, **9**.**72** in the Alpha band, **13**.**44** in the Beta band, and **12**.**62** in the Gamma band. In comparison, the SNR for the raw artifacted signal is 2.32 in Theta, 2.90 in Alpha, 4.33 in Beta, and 0.63 in Gamma. For the DWT-cleaned signal, the SNR is 5.31 in Theta, 8.62 in Alpha, 11.79 in Beta, and 11.43 in Gamma. A general trend where SNR rises with frequency was noted, suggesting that the signal quality improves relative to noise as frequency increases. The graph illustrating these SNR comparisons is plotted in Fig. 7, where orange represents the proposed model’s generated signal, grey represents DWT, and blue represents the raw signal.

Lastly,the SAR for the SAM-40 dataset was calculated. The SAR values are also described in Table 11 alongside SNR. The SAR for the proposed model is **1**.**34**, compared to 1.09 for DWT. This further demonstrates the proposed model’s superior performance in artifact removal and signal preservation.

Hence, the proposed GAN-based denoising model showed remarkable effectiveness in eliminating noise from EEG signal, while keeping the crucial information about brain activity. Quantitative indicators like NMSE, RMSE, and CC show that the model consistently performed well throughout the demanding training and testing process.

## Conclusion

The proposed study offered a unique model “AnEEG” for removing the artifacts from EEG data by utilizing a Generative Adversarial Network-GAN with layers of Long Short-Term Memory-LSTM. EEG is a very essential tool in both neuroscience and clinical fields, providing various brain activities. However, this collected information is always polluted with different unwanted information, which makes it difficult to study brain activity.

By employing GANs with LSTM layers, the proposed model was able to generate artifact-free EEG signals and also successfully preserve essential brain signal data. The quantitative evaluation based on Root Mean Squared Error-RMSE, Normalized Mean Squared Error-NMSE, and Correlation Coefficient-CC highlighted the greater efficacy of the model compared to traditional wavelet-based denoising methods. After evaluation, the model got higher CC and lower NMSE and RMSE values, indicating, stronger linear agreement with the ground-truth signals and better concurrence with the original signals, respectively.

When compared to alternative approaches, some emphasis on specific artifacts, such as ocular artifacts^17^ or motion artifacts^21^, AnEEG effectively handles a broader range of artifacts, including Eye Blink, Eye-movement, Chewing, and Clenching Teeth, across different datasets. This generalizability is essential for real-world applications where multiple artifacts may occur simultaneously. The model consistently outperforms traditional methods like ICA, DWT, and other GAN-based approaches^16^, achieving higher Signal-to-Noise Ratio-SNR and Signal-to-Artifact Ratio-SAR values across different frequency bands and datasets. For example, in the Eye Blink dataset, AnEEG achieves higher SNR in the Beta band (13.08 for GAN-based vs. 10.62 for DWT).

Beyond benchmark datasets, the model has undergone extensive testing on challenging real-world datasets, such as SAM-40, where it continues to outperform existing methods^16^ in SNR and SAR metrics. This adaptability enhances the model’s reliability for practical applications, unlike other models limited to semi-simulated data or specific datasets^19^. Compared to other GAN-based approaches that require extensive training and fine-tuning^15^, AnEEG is more computationally efficient, making it suitable for real-time applications without compromising performance. While other methods like^22^ target single-channel motion artifacts, AnEEG effectively removes multiple artifacts from multi-channel EEG data.

The study in^24^ focused on specific filters and wavelet transforms, which show limitations in detecting short-duration artifacts. AnEEG overcomes these limitations, providing higher CC values and overall better artifact removal performance. Furthermore, methods^13^ that struggle with heavy noise signals, a common issue in real-life environments, are outperformed by AnEEG, which handles challenging artifacts like clenching teeth and chewing more effectively. AnEEG achieves a lower RMSE, with a value of 0.073 in the Eye Blink artifact, compared to 0.076 achieved by the methods in^13^ . Some existing methods required prior knowledge of artifact frequencies^18^, which can be difficult in real-world scenarios. In contrast, AnEEG operates across different frequencies without the need for such specific knowledge. Additionally, while manual selection of noise components^19^ is time-consuming, AnEEG automates the artifact removal process, eliminating the need for manual intervention.

Lastly, whereas a lot of research focuses on limited evaluation metrics, this work employs a comprehensive set of metrics, including RMSE, NMSE, CC, SNR, and SAR, to thoroughly assess AnEEG’s effectiveness in artifact removal.

Overall, the use of deep learning capacity to improve EEG quality yields encouraging results. This advancement delivers more precise brain activity information, facilitating research and clinical diagnosis.

## Data Availability

Downloadable versions of the study’s data are accessible at^34^.
